# T Lymphocytes Expressing AlphaE Beta7 Integrin in Ulcerative Colitis: Associations With Cellular Lineage and Phenotype

**DOI:** 10.1093/ecco-jcc/jjx097

**Published:** 2017-07-17

**Authors:** Christopher A Lamb, John A Kirby, Mary E Keir, John C Mansfield

**Affiliations:** 1 Institute of Cellular Medicine, Newcastle University, UK; 2 Department of Gastroenterology, Newcastle upon Tyne Hospitals NHS Foundation Trust, UK; 3 Research & Early Development, Genentech, USA; 4 Institute of Genetic Medicine, Newcastle University, UK

We thank Dr Horjus Talabur Horje for the comments on T cell lineage commitment in inflammatory bowel disease [IBD] and the role of αE integrin expression on CD8+ T cells. αEβ7 integrin is expressed primarily on T cells in the gut, with 90–95% of αE+ cells in the colon co-staining for CD3.^1^αE+ cells display preferential localisation to the intestinal epithelium, where close interplay exists between leukocytes, epithelial cells, and microbiota.^2,3^

Based on immunohistochemistry, FACS analyses and gene expression of FACS-sorted cells in cohorts of UC patients with reference to control subjects, our study investigated phenotypic differences between αE+ and αE- T cells of both CD4+ and CD8+ lineages.^1^ A striking propensity for Th1, Th17, and Th17/1 phenotype was identified for CD4+αE+ compared with CD4+αE- T cells. The differences between CD8+αE+ and CD8+αE- T cells were limited to a higher gene expression of IL17A and IFNγ by CD8+αE+ compared with CD8+αE- cells, and an increase in IFNγ protein was seen in CD8+αE+ relative to CD8+αE- cells in ulcerative colitis [UC] colonic biopsies. Overall, low levels of IL17A cytokine were expressed by CD8+ T cells, which is consistent with published murine data.^4^ As CD8+αE+ cells are abundant in the gut, higher expression of IFNγ by these lymphocytes may contribute to the inflammation seen in UC.

*Ex vivo* Treg suppression assays were not part of our studies, in part because of limitations on cell numbers obtained from colonic biopsies. However, we did examine expression of the regulatory-associated genes FOXP3, IL10, CTLA4, ICOS, and GARP in T cells sorted by αE expression from control subjects and UC patient colonic biopsies. No difference in expression of any of these genes was observed between CD8+αE+ and CD8+αE- cells. In notable contrast, all five genes were significantly elevated in CD4+αE- compared with CD4+αE+ T lymphocytes in UC, favouring a greater regulatory phenotype in CD4+ cells lacking the integrin and consistent with the pro-inflammatory phenotype we observed in CD4+αE+ cells.

Given the limited discriminating phenotype between CD8+αE+ and CD8+αE- T cells, and the notable differences between CD4+αE+ and CD4+αE- T cells in steady state conditions and during UC, we focused our manuscript on CD4+ T cells.^1^ Corresponding CD8 analyses were also presented. In order to determine the comparative abundance of CD8+αE+ compared with CD4+αE+ T cells, data regarding total colonic T cell expansion in UC, and the relative CD4 to CD8 ratio which in our UC cohort was as high as 6.1:1, must be considered. By gating within the CD3+αE+ cell population, intriguingly we observe that the proportion of CD3+αE+ T cells that were CD4+ and CD8+ was significantly altered between control subjects and UC. The proportion of all αE+ T cells that were CD4+ was 2-fold higher in UC compared with control subjects [Figure 1A], with a corresponding drop in the proportion of the αE+ T cells that were CD8+ [Figure 1B]. Mechanisms controlling the relative frequency, differentiation, and expansion of lymphocytes in the αE+ T cell compartment are worthy of additional exploration to determine if this is a further indication of the role of CD4+αE+ cells in disease pathogenesis.

**Figure 1.**
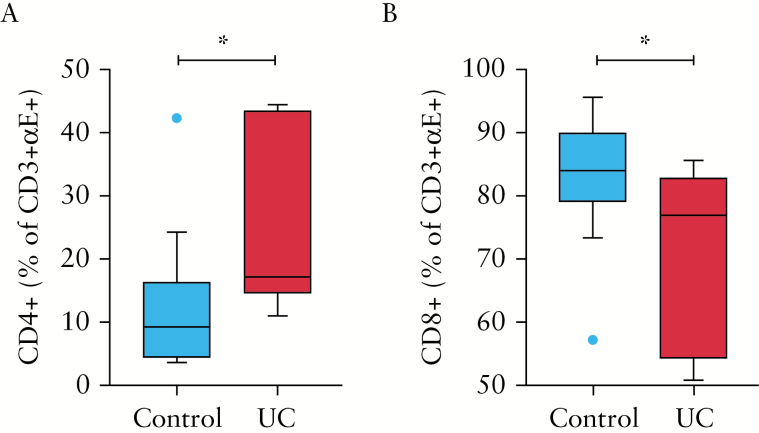
Cells isolated from colonic biopsies sampled from control subjects [*n* = 14] and patients with active UC [*n* = 10] were evaluated by flow cytometry. Frequency of [A] CD4+ and [B] CD8+ cells within the CD3+αE+ subset. Comparisons were determined by unpaired Student’s t test; *p < 0.05. UC, ulcerative colitis.

Within IBD, Crohn’s disease [CD] historically has been considered a Th1-driven disease with higher tissue production of IFNγ than in ulcerative colitis or healthy control subjects.^5^ Since the recognition of Th17 cells in the past 10 years in both CD and UC, this definition has become more complex.^6^ In UC, Th17, Th2, and the recently described subset Th9 have all been identified.^5,7,8^ We described αE+ subsets of Th1, Th17, and Th17/1 cells in UC.^1^ Corroborating the multiplicity of cytokine production associated with these diseases, clinical trials of neither Th1-directed anti-IFNγ, nor Th17-directed anti-IL17A therapy in CD, nor Th2-directed anti-IL13 therapy in UC demonstrated an improvement in clinical response compared with placebo.^9–11^ This observation suggests that neither UC nor CD are defined by a single T helper cell lineage, and highlights the importance of identifying markers of pro-inflammatory cells capable of producing multiple cytokines, in order to better understand disease mechanisms and to develop new targeted therapeutic strategies. In addition to our data highlighting an association of αEβ7 integrin with Th1, Th17, and Th17/1 cells in UC, αEβ7+ T cells in the colon of UC patients have also recently been shown to have a higher potential to produce the signature Th9 cytokine IL9.^1,12^ That the αEβ7 integrin identifies multiple subsets of pro-inflammatory T cells, reinforces the potential importance of these cells in disease pathogenesis and as a potential target for therapy in IBD.

## Funding

This work was supported by the Wellcome Trust [grant number 093885/Z/10/Z to CAL]; by the Biomedical Research Centre of Guy’s and St Thomas’ Hospitals and King’s College London, by the NIHR Newcastle Biomedical Research Centre, and by Genentech Inc. CAL is a Clinical Lecturer supported by the National Institute for Health Research [NIHR].

## Conflict of Interest

CAL: Genentech, Techlab, Immundiagnostik, Roche Tissue diagnostics, Takeda. JCM: AbbVie, Ferring, Genentech, Takeda. JAK: Genentech, GlaxoSmithKline, Intercept Pharmaceuticals. MEK is a full-time employee of Genentech, Inc., a member of the Roche Group.
